# Generation and Characterization of Antibodies against Asian Elephant (*Elephas maximus*) IgG, IgM, and IgA

**DOI:** 10.1371/journal.pone.0116318

**Published:** 2015-02-06

**Authors:** Alan F. Humphreys, Jie Tan, RongSheng Peng, Susan M. Benton, Xiang Qin, Kim C. Worley, Rose L. Mikulski, Dar-Chone Chow, Timothy G. Palzkill, Paul D. Ling

**Affiliations:** 1 Center for Comparative Medicine, Baylor College of Medicine, Houston, Texas, United States of America; 2 Department of Molecular Virology and Microbiology, Baylor College of Medicine, Houston, Texas, United States of America; 3 Department of Molecular and Human Genetics, Baylor College of Medicine, Houston, Texas, United States of America; 4 Human Genome Sequencing Center, Baylor College of Medicine, Houston, Texas, United States of America; 5 Department of Pharmacology, Baylor College of Medicine, Houston, Texas, United States of America; University of Illinois at Urbana-Champaign, UNITED STATES

## Abstract

Asian elephant (*Elephas maximus*) immunity is poorly characterized and understood. This gap in knowledge is particularly concerning as Asian elephants are an endangered species threatened by a newly discovered herpesvirus known as elephant endotheliotropic herpesvirus (EEHV), which is the leading cause of death for captive Asian elephants born after 1980 in North America. While reliable diagnostic assays have been developed to detect EEHV DNA, serological assays to evaluate elephant anti-EEHV antibody responses are lacking and will be needed for surveillance and epidemiological studies and also for evaluating potential treatments or vaccines against lethal EEHV infection. Previous studies have shown that Asian elephants produce IgG in serum, but they failed to detect IgM and IgA, further hampering development of informative serological assays for this species. To begin to address this issue, we determined the constant region genomic sequence of Asian elephant IgM and obtained some limited protein sequence information for putative serum IgA. The information was used to generate or identify specific commercial antisera reactive against IgM and IgA isotypes. In addition, we generated a monoclonal antibody against Asian elephant IgG. These three reagents were used to demonstrate that all three immunoglobulin isotypes are found in Asian elephant serum and milk and to detect antibody responses following tetanus toxoid booster vaccination or antibodies against a putative EEHV structural protein. The results indicate that these new reagents will be useful for developing sensitive and specific assays to detect and characterize elephant antibody responses for any pathogen or vaccine, including EEHV.

## Introduction

Elephant endotheliotropic herpesvirus (EEHV) can cause lethal hemorrhagic disease, particularly in juvenile Asian elephants in captivity [1,2,3,4,5,6] and in range countries [7,8,9]. However, herpesviruses do not typically cause lethal infections in their natural hosts and factors contributing to EEHV-associated disease susceptibility remain enigmatic. The anti-EEHV antibody status of young elephants and normal healthy adults might help to address this issue and the ability to assess elephant immune responses will be needed to evaluate future EEHV vaccines. To our knowledge, specific serology assays for the detection of anti-EEHV antibody titers in Asian elephants have not been reported. Previous studies in African savanna elephants and Asian elephants indicated that both species appear to make IgG [10,11] but these studies failed to confirm the presence of IgM or IgA. Readily available reagents for the detection of elephant IgG are also limited [10,12,13]. A more recent study characterizing the genomic organization of African savanna elephant immunoglobulin genes indicates that in addition to IgG, African savanna elephants encode for a μ constant region [14]. To our knowledge, there is no publicly available sequence information for either Asian or African savanna elephant α constant regions. Very little specific information is known regarding immunoglobulin genes in other members of the Afrotheria superorder, although there has been specific antisera generated towards IgG in manatees (*Sirenia*) and identification of cross-reacting commercial antisera towards IgG in elephant shrews (*Macroscelidea*) and hyraxes (*Hyracoidea*) [15,16]. In order to address some gaps in our understanding of elephant immunoglobulin isotypes, we sought to determine whether Asian elephants produce IgM or IgA and to use this information to generate or identify specific antibodies that could detect these immunloglobulin isotypes. Mice were also immunized with Asian elephant IgG prepared from serum to generate a specific anti-elephant IgG monoclonal antibody. These reagents were used to determine whether Asian elephants produce these immunoglobulin isotypes in blood and milk and to assess antibody responses following booster vaccination with tetanus toxoid or to detect anti-EEHV antibodies to a putative EEHV glycoprotein.

## Materials and Methods

### Animals and sample acquisition

Serum and milk samples were acquired from a 33-year old female Asian elephant at the Houston zoo. Tetanus booster vaccination and serum collection were carried out as described previously [13]. Serum samples used for the anti-gL ELISA assays were from a 20-year old female and a 4-year old male, both located at the Houston zoo. Institutional Animal Care and Use Committees at Baylor College of Medicine and the Houston Zoo reviewed and approved the research described in this study.

### PCR of Asian elephant genomic DNA

Primers 5’-TCAGCTCTGCCCTGACA-3’ and 5’-GAAGCAGGTATTGGCCGTGTC-3’ were designed based on the African savanna elephant μ constant region and were used to PCR amplify the μ constant region encoding exons CH1–4 from Asian elephant DNA [17] that had been analyzed previously. PCR products of 1965bp from two independent amplifications were sequenced using standard Sanger sequencing methods and found to be identical to each other and are 99% identical to the African savanna elephant (S1 Fig.). The sequence file has been deposited in GenBank under the accession number KJ567049.

### Purification and protein sequencing elephant serum IgA

Asian elephant IgA was purified with SSL7 agarose (Invitrogen) according to the manufacturer’s protocol. The eluted fractions were analyzed by SDS-PAGE and coomassie staining. A polypeptide of approximately 55–60kDa was detected and subjected to tandem mass spectrometry as described previously [18].

### Generation of anti-IgM antibodies

**Fig 1:**
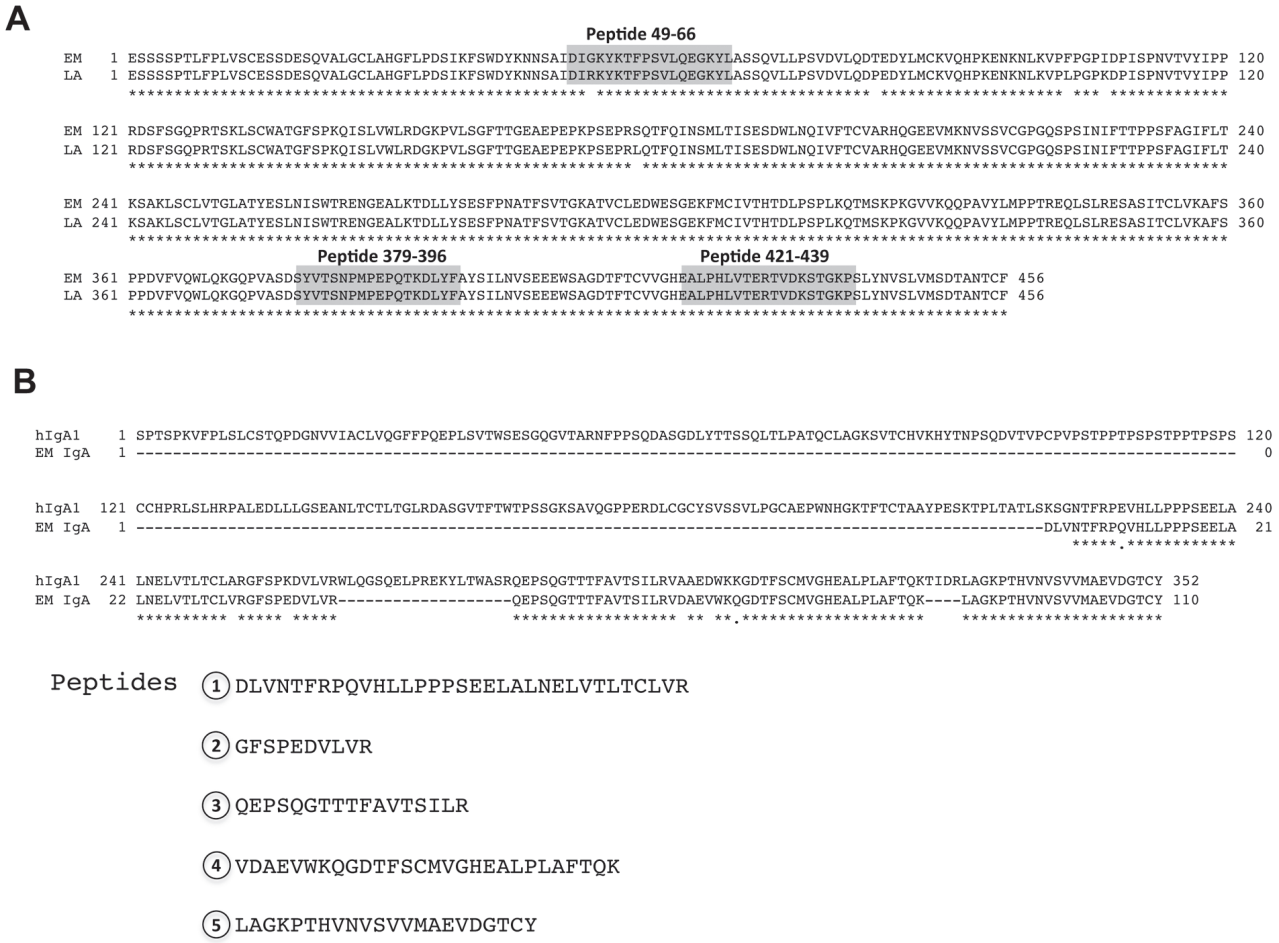
Predicted amino acid sequence of African savanna elephant and Asian elephant IgM and partial sequence of Asian elephant IgA. A) Alignment of Asian elephant (EM) and African savanna elephant (LA) IgM heavy chain constant region amino acid sequences (CH1–4) predicted from genomic sequencing. Peptide sequences used to generate elephant specific anti-IGM antibodies are shown outlined in grey shading. B) Alignment of human (hIgA1) and partially determined Asian elephant IgA protein sequences that were assembled from 5 peptides. Following tandem mass spectrometry analysis of a partially purified Asian elephant IgA, five peptides were identified and are listed below the aligned sequences.

Kyte-Doolittle Hyrophobicity and Hopp-Woods Hydrophilicity analysis of the predicted Asian elephant constant region of IgM was used to identify 3 antigenic peptides (Fig. 1). The peptides were synthesized and conjugated with KLH. Two rabbits each were inoculated with each peptide. Rabbits were inoculated and then boosted at days 14, 42, and 56. The rabbits were bled at day 72 and following confirmation that they had high titers towards the peptide were terminally bled at day 103. Approximately 50ml of each peptide specific serum was affinity purified using the same peptide used for immunization and then conjugated with HRP using the EZ-Link plus activated peroxidase kit (Thermo Scientific, Rockford, IL).

### Generation of anti-IgG monoclonal antibody

Asian elephant IgG was purified by Protein A/G affinity chromatography (Pierce) and concentrated with a 9K MWCO Pierce Concentrator (Thermo Scientific, Rockford, IL) to 2mg/ml. The final protein preparation was dialyzed into PBS. Female BALB/c mice (6–8 weeks of age, Jackson Laboratories, Inc., Bar Harbor, ME) were injected with purified elephant IgG as an emulsion in Freund’s Complete Adjuvant (Sigma, St. Louis, MO). The primary injections were given s.c. with 100 μg of elephant IgG. Two booster injections were given at two-week intervals with 50 ug of elephant IgG as an emulsion using Incomplete Freund’s Adjuvant, alternating between s.c. and i.p. routes of injection. At 3 days before cell fusion, one selected mouse was injected i.p. with 50 ug of elephant IgG prepared in PBS. Sera from immunized mice were tested for antibody titers by ELISA. ELISA 96-well plates (Immunolon II, Dynatech Corp., Chantilly, VA) were coated with 10 μg/ml of purified elephant, rabbit, or human IgG. ELISA plates were blocked with 1% BSA and incubated overnight at 4°C with antisera at different dilutions, and plates were developed with secondary goat-anti mouse IgG-horseradish peroxidase conjugated antibody. Antisera from the mice were also assayed by Western blot and the mouse with the best profile was chosen for the fusion. A fusion between mouse spleen cells and the mouse SP2/0-Ag14 myeloma cell line (purchased from ATCC) was performed using standard PEG fusion methodology. Hybridomas were screened by ELISA for antibodies to elephant IgG. Those showing a strong ELISA reaction were screened again by ELISA to elephant, rabbit, and human IgG. Positives from the second ELISA assay were screened by Western blot for specificity to elephant IgG heavy chain. One clone (516 G6) was selected that only reacted with heavy chain and was further cloned by limiting dilution. The monoclonal antibody was then conjugated with HRP using the EZ-Link plus activated peroxidase kit (Thermo Scientific, Rockford, IL)

### Gel filtration of Asian elephant serum

Gel filtration was conducted using an AKTA FPLC system (GE healthcare Life Sciences, Pittsburgh, PS, USA). Briefly, 0.5ml serum or milk (milk was pre-centrifuged at 20,000g to remove insoluble material) from a healthy adult Asian elephant was filtered through a 0.22uM filter (Millipore, Billerica, MA, USA) and then centrifuged for 30 minutes at 20,000g. Serum or milk samples were then applied to a Superdex 200 10/300 GL column that had been equilibrated in phosphate buffered saline (PBS) and then run with a flow rate of 0.5ml/minute. Fractions were collected and analyzed by Western blot. To determine the approximate molecular sizes of proteins eluting off the column, separate identical runs were conducted with the following standards: Adolase (176kDa), Catalase (219kDa), Ferritin (416kDa), and Thyroglobulin (699kDa), or Blue Dextran 2000 (2000kDa) and Ribonuclease A (13.7kDa).

### Antibodies and ELISA assays

Heavy-chain specific goat anti-human IgA-HRP was purchased from InvivoGen (San Diego, California, USA). ELISA assays for detection of anti-tetanus toxoid or anti-EEHV1A gL antibodies were carried out as described previously [13]. Briefly, ninety-six well polystyrene plates (Immulon 4 HBX, Thermo Electron Corp., Milford, MA) were coated with either 1 ug of purified tetanus toxoid (List Biological Laboratories,US) at a 20 ug/mL concentration in Carbonate Bicarbonate Buffer (pH 9.6) or 100ng of purified recombinant EEHV1A gL at 2 ug/ml, also in Carbonate Bicarbonate Buffer (pH 9.6). Negative control wells consisted of no protein coating and 1 ug of BSA. The plate was then incubated overnight at 4°C, washed with PBS/0.5% Tween, and blocked with 1% teleostean gelatin-PBS (Sigma Chemical Co., U.S.) for 1 h at 37°C. The plates were then blocked for 2 hours at RT with blocking buffer (5% non-fat dry milk as in 2.5g non-fat dry milk powder in 50 ml 0.01 PBS) and washed twice with PBS/Tween 0.05%. Elephant serum samples were serially diluted two-fold in blocking buffer. Tetanus toxoid or gL-specific IgG was detected using the horse radish peroxidase (HRP)-conjugated mouse monoclonal anti-elephant-IgG. The ELISA was developed by addition of 3,3’,5,5’-tetramethybenzidine substrate (TMB Microwell Peroxidase Substrate System, Kirkegaard & Perry Laboratories Inc., U.S.) and the reaction was stopped by the addition of H_3_PO_4_. The A450 of each well was determined spectrophotometrically with an ELISA reader (EE 311SX Microplate Autoreader, Bio-Tek Instruments, USA). Values with an OD>0.05 were considered positive. The antibody titer was defined as the reciprocal of the highest dilution resulting in an OD>0.05. Negative cutoff was established as the mean OD of the negative samples plus 3 standard deviations.

### Recombinant EEHV gL protein

Predicted outer surface glycoprotein (gL) was cloned utilizing information from the complete genome sequence of EEHV1A [17]. The following oligonucleotides were used to PCR amplify the gene: forward primer 5’-ATGATCACAAATGTAAATTTGATGTACGGT-3’; and reverse primer, 5’-CCCACCGGGTTGAGATACT-3’. DNA from the necropsy tissue of a fatal EEHV 1A case [17] was combined with the oligonucleotides and amplified and cloned into PCR-blunt (Invitrogen, Waltham, MA USA) according to the manufacturers instructions. The plasmid was checked by sequencing to ensure no errors were introduced during PCR and then the gene was subloned into PET28a (Clontech, Mountain View, CA, USA). Recombinant proteins were expressed by induction with IPTG and soluble protein was purified using NTA-agarose beads as described previously [19].

## Results

### Determination of the genomic coding region for the Asian elephant μ constant region and partial sequencing of purified serum IgA

We recently determined the complete genome sequence of EEHV1A from a 12-year-old hemorrhagic disease case [17]. During this process, approximately twenty-two gigabases of raw sequence data reads were produced from the Asian elephant genome, providing sufficient information to determine Asian elephant gene sequences by comparison with the African savanna elephant genome sequence. To identify genomic sequences that might encode Asian elephant IgM, Burrows-Wheeler Aligner (BWA) was used to align the available Asian elephant genome sequence data to the African savanna elephant elephant reference genome (LoxAfr3). All of the matched reads were then put into a combined file and a blast search was performed with the Asian elephant contigs against the African savanna elephant genome to identify matches with the IgM constant region. One of the derived contigs was nearly identical to the hypothetical CH3 and CH4 u-constant region from the African savanna elephant (*Loxodonta africana*) [14], which was retrieved from the UCSC genome browser (http://genome.ucsc.edu/) (data not shown). PCR primers were designed to amplify the entire genomic coding region for the μ constant region from Asian elephant DNA based on the African savanna elephant sequence, due to its apparent close similarity in this region. The resulting PCR products were sequenced and found to differ by only 13 bases over 1965bp (99% identical) (S1 Fig.). The putative Asian elephant μ constant region is 99% identical to the African savanna elephant over 465 amino acid residues (Fig. 1A).

To our knowledge, there is no publicly available genomic sequence information for the α constant region of IgA from African savanna or Asian elephants. To determine whether Asian elephants produce IgA, we used SSL7 (*Stapholococcus aureus* superantigen-like protein 7) chromatography to purify Asian elephant IgA from serum. SSL7 has been shown previously to bind with high affinity to monomeric forms of human, pig, rat, and horse IgA and has no affinity for human IgG. Purified material was analyzed by SDS-PAGE and coomassie staining (data not shown). A polypeptide migrating with approximate molecular weight of 55–60 kDa, which corresponds to the average size of the IgA heavy chain, was extracted from the gel and subjected to tandem mass spectrometry. Several large peptides were generated that aligned with the carboxy-terminal third of human IgA (Fig. 1B).

### IgG, IgM, and IgA are detectable in Asian elephant serum and milk

**Fig 2:**
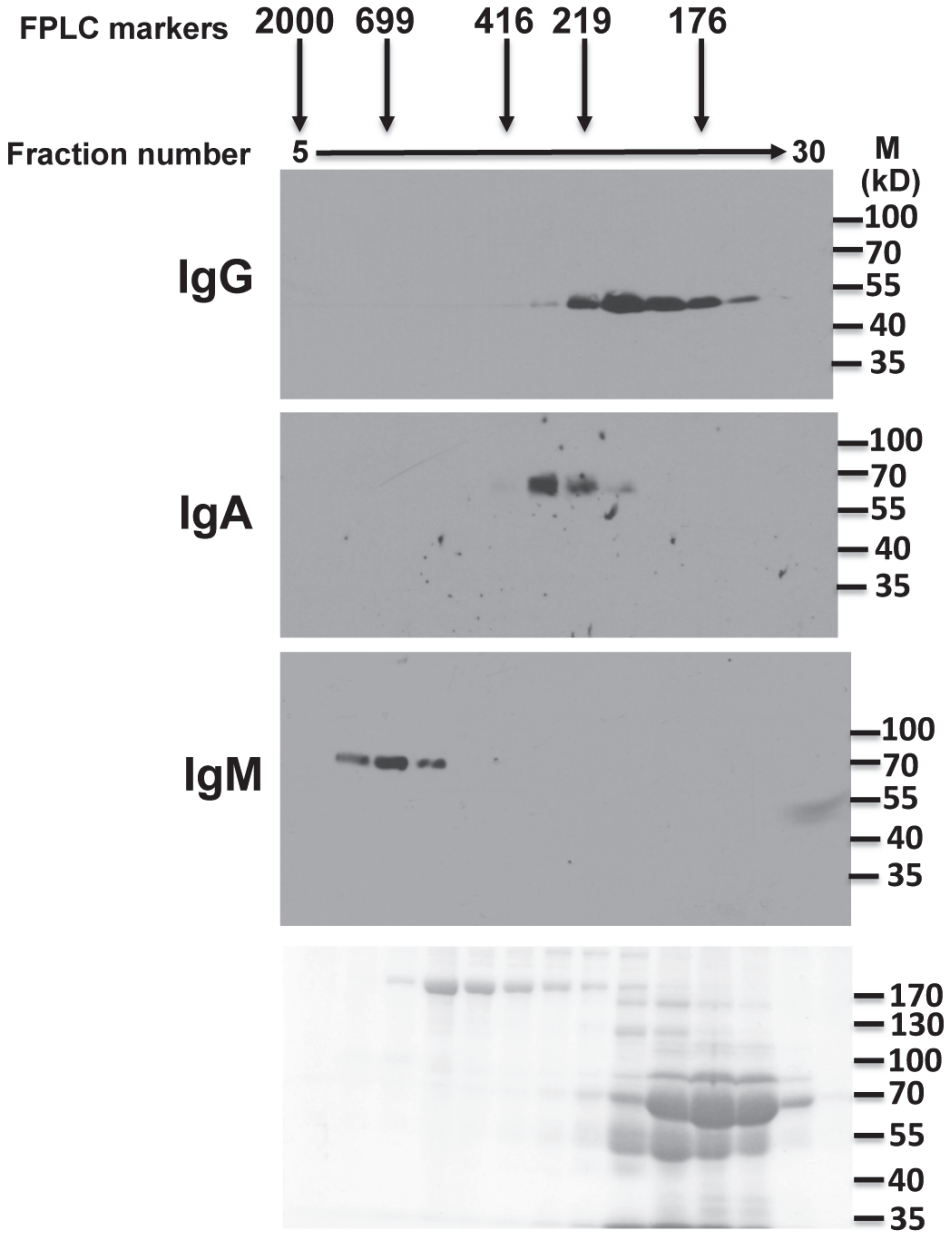
Immunoblot analysis of Asian elephant serum following fractionation by gel filtration. Liquid fractions eluting off from gel filtration of Asian elephant serum were resolved and analyzed by immunoblotting. Fraction numbers are labeled at the top and the approximate sizes of marker proteins are indicated. Three identical gels were then probed with isotype specific antisera or monoclonal antibody (anti-IgG) as indicated to the left of each blot. The bottom panel is a coomassie stained gel identical to the gels used for the Westerns above. Molecular weight markers for the blots are shown on the right of each panel.

**Fig 3:**
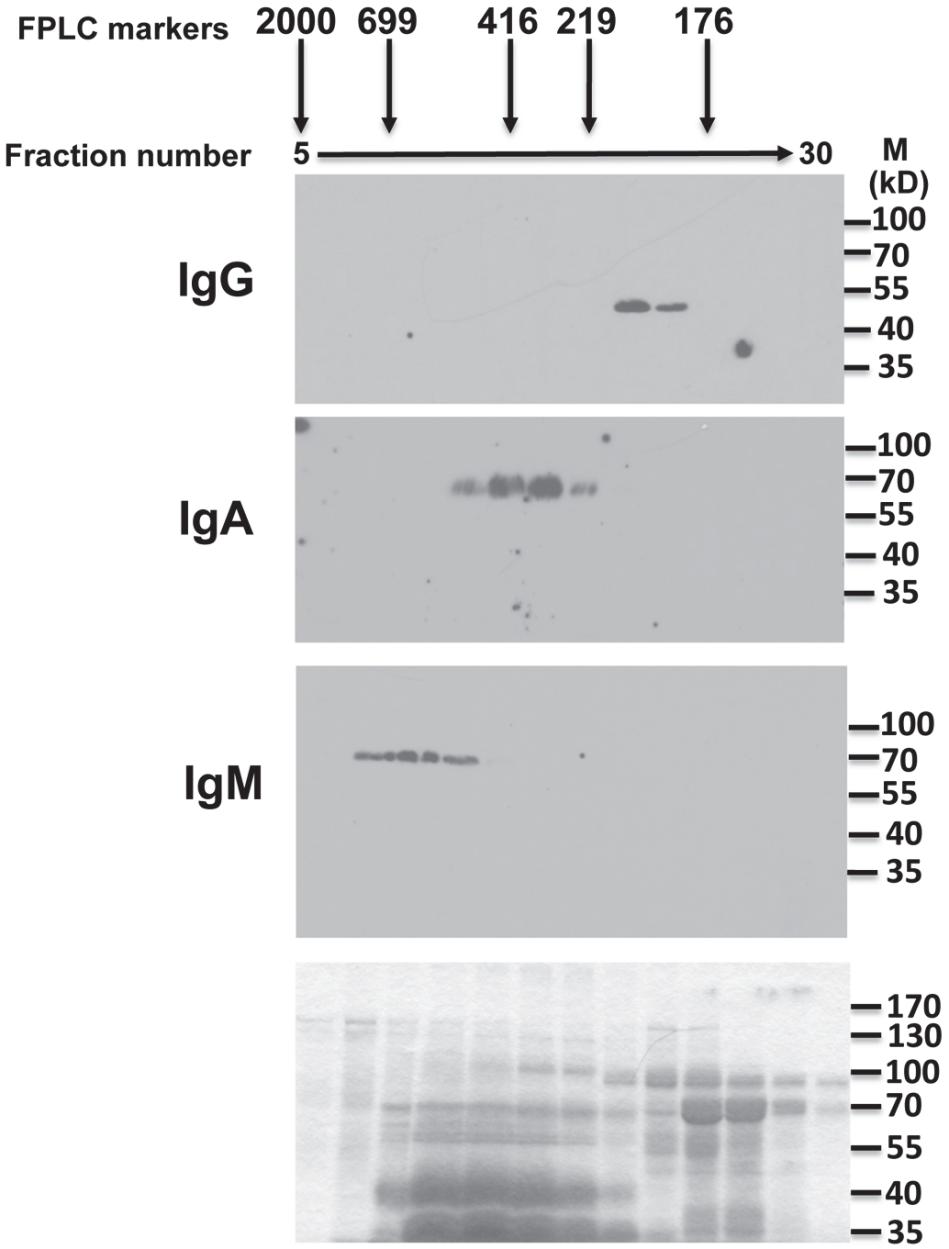
Immunoblot analysis of Asian elephant milk following fractionation by gel filtration. Liquid fractions eluting off from gel filtration of Asian elephant milk were resolved and analyzed by immunoblotting. Fraction numbers are labeled at the top and the approximate sizes of marker proteins are indicated. Three identical gels were then probed with isotype specific antisera or monoclonal antibody (anti-IgG) as indicated to the left of each blot. The bottom panel is a coomassie stained gel identical to the gels used for the Westerns above. Molecular weight markers for the blots are shown on the right of each panel.

**Fig 4:**
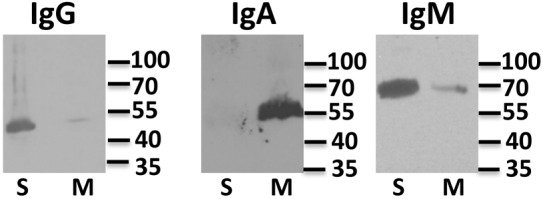
IgG, IgA, and IgM are present in Asian elephant milk and serum. Equal volumes of serum and milk were resolved by SDS-PAGE and then analyzed by immunoblot. Each blot was probed with antibodies specific for Asian elephant IgG, IgA, or IgM as indicated above each panel. Lanes containing serum (S) and milk (M) are labeled below each panel. Molecular weight markers are indicated to the right of the panels.

To confirm that Asian elephants produce IgM, we synthesized three potential immunogenic peptides based on the putative IgM constant region protein sequence in Fig. 1A. The peptides were used to generate specific antisera in rabbits. In addition, since at least part of the Asian elephant IgA protein sequence was similar to human IgA, we hypothesized that commercially available anti-human IgA antibodies might cross-react with Asian elephant IgA. Finally, we also generated a monoclonal antibody to the heavy chain of Asian elephant IgG. These antibodies were used to detect the three major immunoglobulin isotypes in elephant serum following gel filtration (Fig. 2). IgM, IgA, and IgG are expected to elute from the column at approximately 900 kDa, 170 kDa, and 150 kDa respectively. In addition, the heavy chains of each isotype are approximately 70 kDa, 55 kDa and 50 kDa respectively when analyzed by SDS-PAGE. As expected, each of the specific antisera or monoclonal antibodies detected serum polypeptides that eluted from gel filtration in the expected order and resolved by SDS-PAGE according to their expected sizes (Fig. 2). No cross-reactivity was observed, demonstrating specificity for each of the antibodies. Anti-sera to all three IgM peptides also gave similar results (data not shown). Asian elephant serum subjected to either protein A/G, mannan binding protein, or SSL7 affinity chromatography yielded partially purified preparations of IgG, IgM, and IgA that were only recognized by the specific corresponding antibodies for each of the immunoglobulins, further confirming the specificity of the antibodies (data not shown). Gel filtration of milk gave similar results (Fig. 3), although IgA eluted slightly earlier than seen in serum, suggesting that a proportion exists in a polymeric or dimeric form that might be expected with the secreted form of this immunoglobulin isotype. Having confirmed that Asian elephants produce each of the major immunoglobulin classes in serum and milk, we used our specific antisera to determine their relative proportions in these fluids. Equal volumes of serum and milk were subjected to SDS-PAGE and immunoblots were carried out with each of the isotype specific antibodies (Fig. 4). IgG levels in serum are substantially larger than in milk. Conversely, IgA levels in milk are substantially larger than found in serum. Serum IgM is somewhat more abundant than in milk. At the current time, we are unable to make accurate judgments about the actual concentrations of any of the immunoglobulin isotypes in serum or milk because the fractions from the gel filtration experiments contain multiple polypeptides or the polypeptides are at low levels and not visible by coomassie staining (Figs. 2B and 3B). All milk samples tested from a 3 month time period following tetanus booster vaccination showed similar results as those shown in Fig. 4 (data not shown).

### Detection of elephant antibodies to tetanus toxoid and EEHV gL

**Fig 5:**
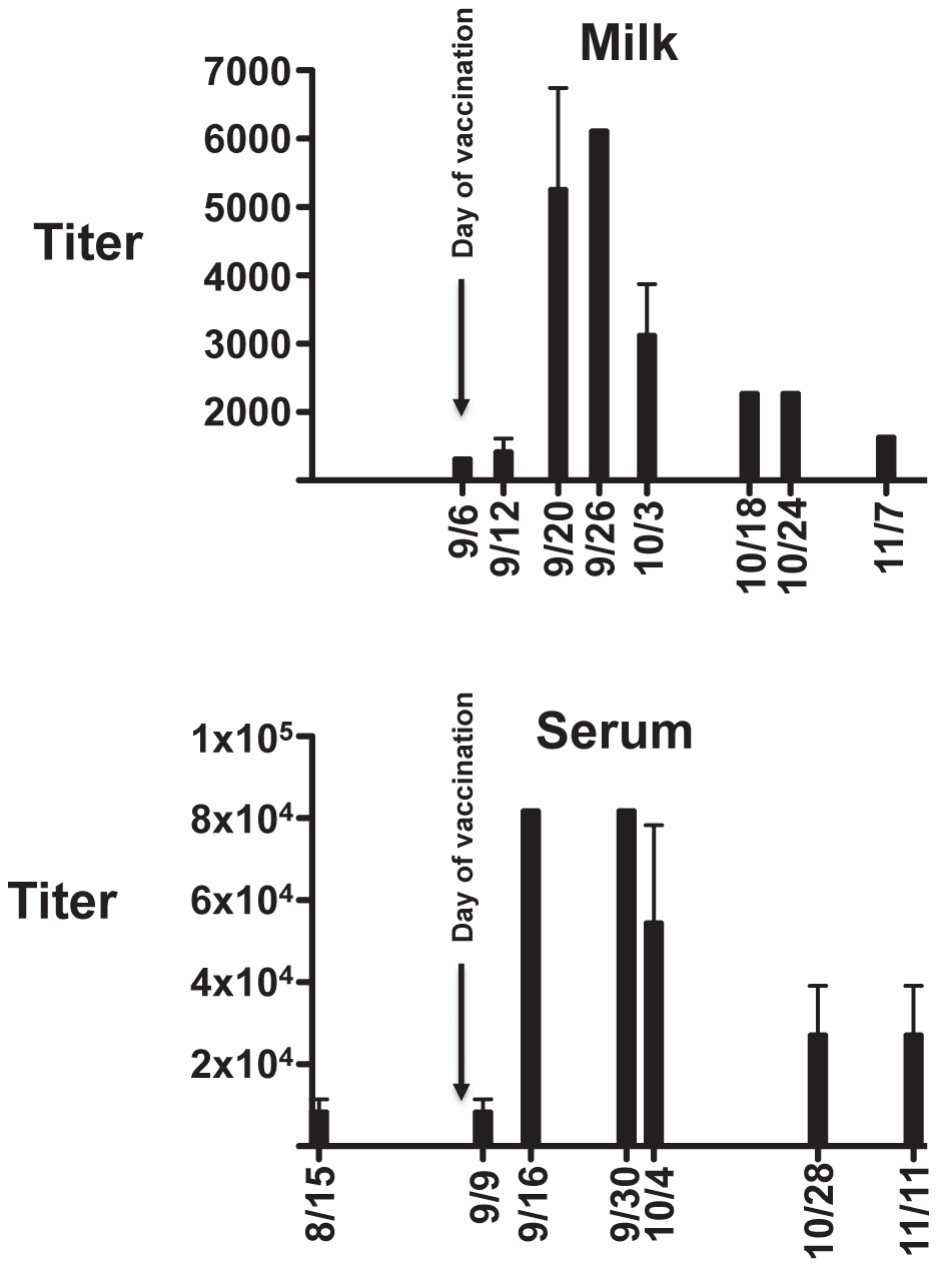
Anti-tetanus toxoid IgG titers in milk and serum following booster vaccination with tetanus toxoid. The graphs show anti-tetanus IgG antibody titers (y-axis) for milk and serum from an adult female Asian elephant. Samples were tested on days indicated below the x-axis. The results are shown as the mean plus standard deviations from three independent experiments.

**Fig 6:**
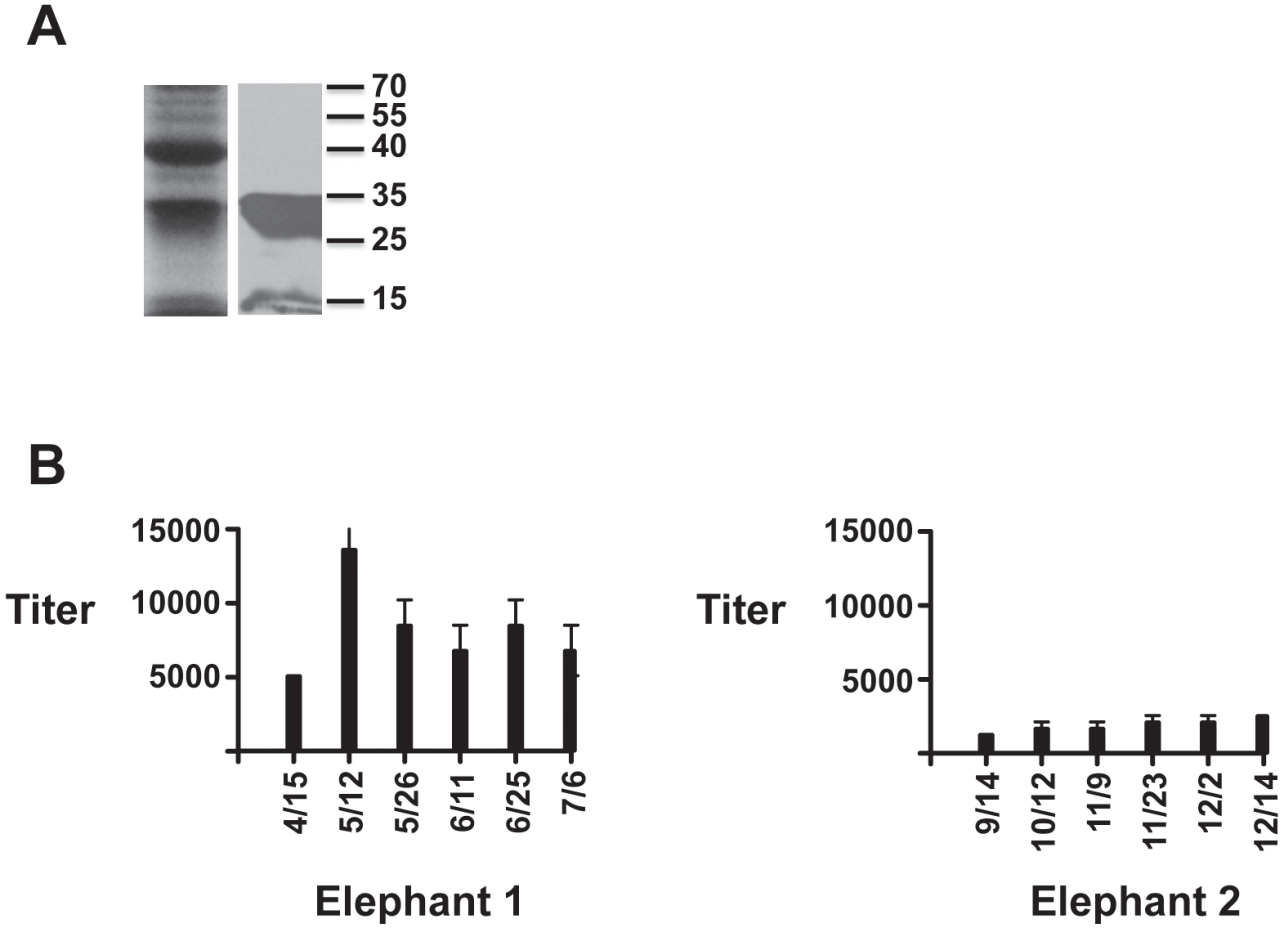
Anti-EEHV1 gL serum titers in two elephants with documented EEHV viremia and shedding. A) Recombinant EEHV gL expressed in e. coli and purified using metal affinity chromatography. The panel on the left is a coomassie stained gel of purified gL protein and the panel on the right is the same protein detected in an immunoblot using an anti-histidine antibody. Molecular weight markers are indicated on the right. B) The graphs show anti-gL IgG antibody titers (y-axis) for serum from an adult female Asian elephant (elephant 1) and a juvenile male elephant (elephant 2). Samples were tested on days indicated below the x-axis. The results are shown as the mean plus standard deviations from three independent experiments in which each independent experiment had four replicates for each dilution.

To further test the utility of our anti-elephant immunoglobulin antibodies we looked at antibody titers following routine booster vaccination of an elephant with tetanus toxoid. Sequential milk and serum samples were obtained from a 33-year old female elephant that was currently nursing her three-year old calf following tetanus booster vaccination. As expected, serum anti-IgG titers to tetanus toxoid rose approximately 8-fold 2–3 weeks following booster vaccination and fell to almost baseline levels within 90-days post-vaccination (Fig. 5). Interestingly, a similar pattern was detected in milk from the same animal, although absolute titers were 10–15 fold lower relative to serum titers (Fig. 5). No antibody titer changes were detectable in serum or milk for IgM or IgA following tetanus booster vaccination (data not shown). In addition to tetanus toxoid, we wanted to use our newly characterized reagents to establish a first generation ELISA assay to detect anti-EEHV antibodies. Conserved herpesvirus glycoproteins, particularly gB and gH, have frequently been used to establish such assays, but so far we been unable to successfully express the EEHV gB and gH homologs (data not shown). However, we did achieve robust expression of gL (Fig. 6). Due to the lack of any serological assay for EEHV1, we decided to use this protein to set up a first generation ELISA assay to detect anti-EEHV antibodies. Serum from two animals was tested who were known to have been previously infected with EEHV1 [20]. Anti-EEHV gL titers ranged from 5000–10,000 for the adult female elephant and were slightly lower in the juvenile male, ranging from 1200–2400 (Fig. 6). Over a 3-month period for each elephant, the titers remained relatively constant. Like the tetanus toxoid assays, no detectable IgM or IgA anti-gL titers above background were observed (data not shown).

## Discussion

The results from this study indicate that Asian elephants produce IgM and IgA in both serum and milk. To our knowledge, this is the first evidence providing confirmation that Asian elephants produce these immunoglobulin isotypes and the first evidence for the presence of all three immunoglobulin isotypes (IgG, IgA, and IgM) in elephant milk. We also generated a monoclonal anti-elephant IgG antibody, which was successfully used to monitor IgG levels in serum and milk following booster vaccination with tetanus toxoid. In addition, we found detectable antibodies reactive against the EEHV glycoprotein gL. While none of these observations are surprising, the information and reagents generated by our study are new, and will be required for conducting more detailed antibody responses in endangered Asian elephants, including those against EEHV.

While our newly developed reagents were sufficient to confirm the presence of IgM and IgA in serum and milk, they did not detect any response following tetanus toxoid vaccination or in the gL ELISA assay. The adult female elephant studied in this investigation has been routinely booster vaccinated with tetanus toxoid for several years as part of her normal health program. Therefore, the lack of an anti-tetanus toxoid IgM response, which is usually stimulated following primary exposure to antigen, was not surprising. Similarly, both elephants who were tested in the gL ELISA had previously documented EEHV-1 associated viremias so no anti-IgM specific EEHV1A reactivity was expected. Typically, IgA responses are generated towards mucosal antigens and the tetanus toxoid vaccine is administrated intramuscularly, so it is not surprising that no detectable IgA response is generated with this vaccine. While EEHV1 is thought to be spread through nasal secretions and might be expected to induce some secretory IgA response, we did not have sufficient milk to test this possibility in the gL ELISA. In addition, levels of IgA appear to be quite low in serum (Fig. 4) and this may have also contributed to the inability to detect any serum IgA antibodies to gL. However, we cannot exclude an explanation in which other elephants might have detectable IgM or IgA responses under similar circumstances and individual variations (e.g., genetic) contributed to undetectable isotype responses from these particular animals.

While our reagents were useful for confirming that Asian elephants produce all three major immunoglobulin subtypes, these reagents have some potential limitations that are important to consider. First, the number of IgG subclasses in Asian elephants remains unknown. African savanna elephants possibly encode up to 8 IgG subclasses [14], but how many are functional or produced is unknown. Whether Asian elephants encode a similar array of IgG subclasses is also unknown and how many of these are detected by the monoclonal used in the present study is unclear. At least for the tetanus toxoid ELISA, we found that the monoclonal anti-IgG was about 2–4 fold more sensitive than a rabbit polyclonal anti-Asian elephant IgG used in a previous study [13] (data not shown), so it appears to detect at least a significant proportion of anti-tetanus IgG antibodies. In some species multiple IgA subclasses also exist, but at present, our protein sequencing data did not detect any apparent variations. Finally, while we can deduce the relative levels of an individual immunoglobulin isotype when comparing equal volumes of milk and serum, we are not able to determine the relative levels of IgG to IgA or IgM because the affinities of each of our specific detection reagents for these isotypes remains unknown. Additional studies in which more highly purified preparations of these immunoglobulin isotypes are achieved or the relative affinities or sensitivity or our detection reagents is determined will be needed to quantitate their absolute abundance in serum or milk. Another limitation is that we only assessed relative levels of the immunoglobulin isotypes in milk from a limited time period. It’s quite possible that immunoglobulin isotype levels may fluctuate in milk depending on the post-pregnancy time the milk samples are acquired. Future studies will need to focus on validating whether or not Asian elephants encode the equivalent IgG subclasses found in African savanna elephants, and how many of them are detected by the monoclonal antibody used in this current study. Similar studies will be needed to determine whether or not multiple α constant regions are also present and to assess milk immunoglobulin levels over a longer period beginning with the birth of a calf.

## Supporting Information

S1 Fig Alignment of genomic sequences from Asian (EM) and African savanna elephant (LA) encoding four exons and three introns of the IgM constant region. Exons are shaded in grey and labeled to the right of the sequence alignment. The sequence for the African savanna elephant IgM constant regions CH1–4 was retrieved from the UCSC genome browser (http://genome.ucsc.edu/) and the homologous sequence from the Asian elephant has been deposited under accession number KJ567049.
(TIF)
