# Supplementary figures and images for: Generation and Characterization of Antibodies against Asian Elephant (Elephas maximus) IgG, IgM, and IgA
Source: PLoS One. 2015 Feb 6;10(2):e0116318. doi: 10.1371/journal.pone.0116318 (PMC4320114; doi:10.1371/journal.pone.0116318)

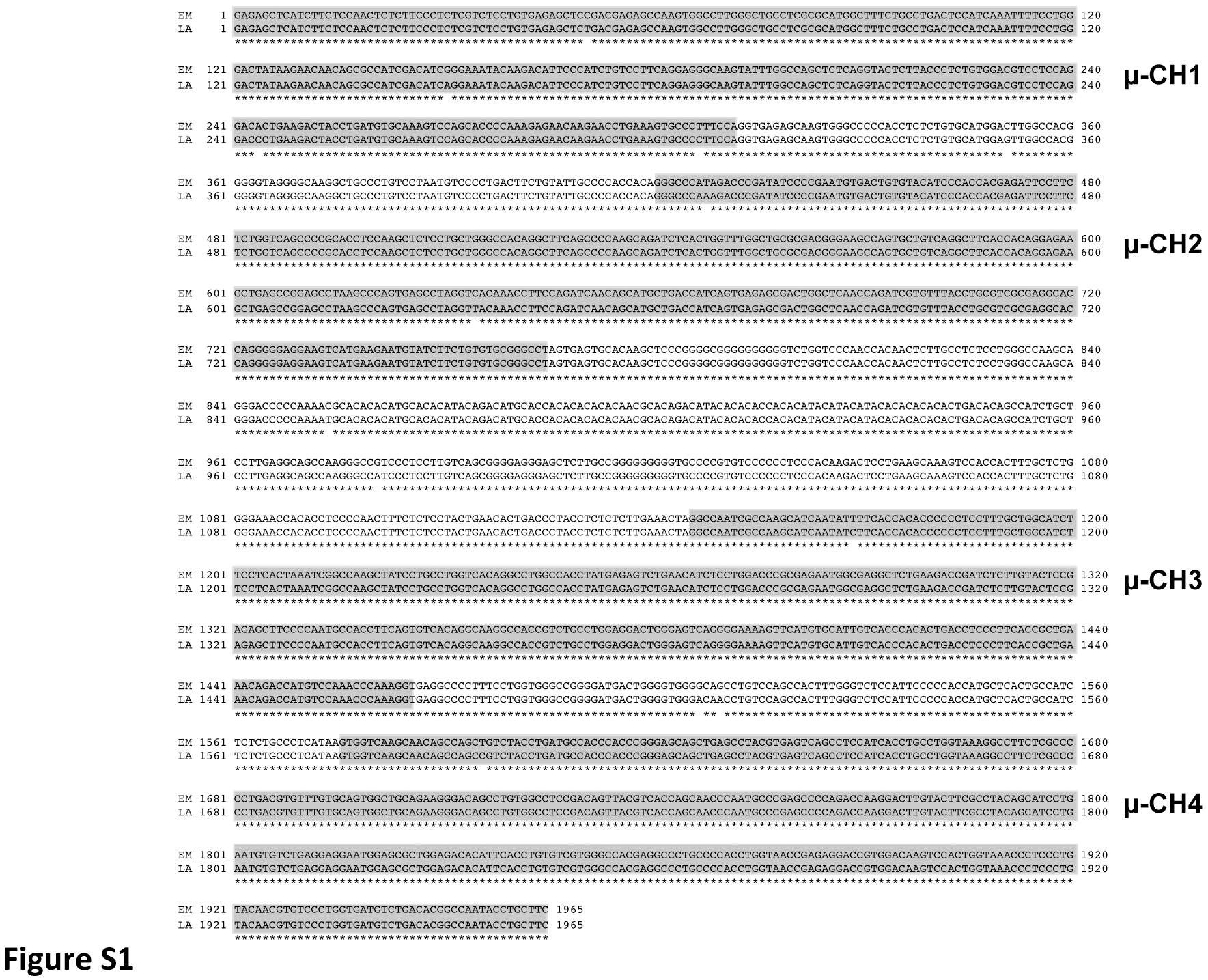

Supplement: S1 Fig — Exons are shaded in grey and labeled to the right of the sequence alignment. The sequence for the African savanna elephant IgM constant regions CH1–4 was retrieved from the UCSC genome browser (http://genome.ucsc.edu/) and the homologous sequence from the Asian elephant has been deposited under accession number KJ567049. (TIF) [file pone.0116318.s001.tif]
